# Acknowledgement to Reviewers of *Toxins* in 2014

**DOI:** 10.3390/toxins7010043

**Published:** 2015-01-08

**Authors:** Toxins Editorial Office

**Affiliations:** MDPI AG, Klybeckstrasse 64, CH-4057 Basel, Switzerland

The editors of *Toxins* would like to express their sincere gratitude to the following reviewers for assessing manuscripts in 2014:

Aas, Pål
Abbès, Samir
Abee, Tjakko
Abou-Mansour, Eliane
Abraham, Ann
Abrunhosa, Luís
Adamo, Roberto
Adang, Michael J.
Akhtar, Yasmin
Aktories, Klaus
Albrecht, Eric A.
Alderman, Stephen
Ali, Syed Abid
Alonso, Juan Carlos
Ammons, David
Amuzie, Chidozie J.
Anangi, Raveendra
Anderson, George
Anderson, Peter D.
Antonissen, Gunther
Apone, Fabio
Appelbaum, Lior
Arbillaga, Leire
ARGILES, Angel
Argilés, Àngel
Arias, Renée S.
Ariga, Hiroyoshi
Arimitsu, Hideyuki
Asakawa, Manabu
Atapattu, Dhammika
Audenaert, Kris
Avantaggiato, Giuseppina
Avanzini, Giuliano
Bailly, Jean-Denis
Bakke, Merete
Balsinde, Jesús
Bammens, Bert
Barra, Donatella
Barrientos Velazquez, A.L.
Barrington, Dani
Basak, Ajit K.
Basti, Leila
Baxa, Dolores V.
Beddoe, Travis
Behle, Robert
Belibasakis, Georgios N.
Ben-Dov, Eitan
Benoit, Joshua
Bensmail, Djamel
Bergstrom, Gary C.
Bernhoft, Aksel
Berrada, Houda
Berry, Colin
Berry, John P.
Berthiller, Franz
Bertuzzi, Terenzio
Bevilacqua, Antonio
Bideshi, Dennis
Billiald, Philippe
Bishop, Alistair
Blasi, Juan
Boles, Blaise R.
Bonn, Gunther K.
Börjesson, Thomas
Bosilevac, Joseph M.
Bosman, Giel
Bosmans, Frank
Botana, Luis
Bouaïcha, Noureddine
Bougis, Pierre
Boulogne, Isabelle
Bourhis, Xuefen Le
Bradley, Ken
Bradshaw, Rosie
Braga, Thiago Verano
Brand, Larry
Brantl, Sabine
Bresciani, Mariano
Briner, Wayne
Bröker, B.M.
Brooks, Samantha A.
Brown, Robert
Brunetti-Pierri, Nicola
Bucciarelli, Gary M.
Büchs, Wolfgang
Bugg, Timothy D.H.
Bulla, Lee A.
Burtey, Stephane
Butrón, Ana
Buttenschoen, Klaus
Calvete, Juan J.
Campos, Alexandre
Camu, William
Capell, T.
Casewell, Nicholas R.
Casiraghi, Federica
Castagnola, Anaïs
Castiglioni, Bianca
Castoe, Todd
Caudle, Robert
Cavaliere, Chiara
Chae, Han-Jung
Chan, Thomas
Chang, Long-Sen
Chang, Perng-Kuang
Cheli, Federica
Chen, Rong
Chen, Tianbao
Chen, Yau-hung
Cheng, Luisa W.
Chichger, Havovi
Chippaux, Jean-Philippe
Chitalia, Vipul
Chooi, Yit-Heng
Chua, Mei-Sze
Chung, Shin-Ho
Ciccoli, Lucia
Claus, Harald
Cohen, Gerald
Coronel, M.B.
Corver, Jeroen
Crickmore, Neil
Croubels, Siska
Culmsee, Carsten
Daines, Dayle
Dänicke, Sven
Davies, Peter L.
De Girolamo, Annalisa
De Graaf, Dirk
De Haro, Luc
De Pedro, Nuria
De Rijk, Theo C.
Degen, Gisela H.
Delcour, Anne
Deng, Youjun
DeRosa, Maria C.
Di Maro, Antimo
Di, Rong
Diaz-Orejas, Ramon
Diehl, Sean
DiRienzo, Joseph M.
Doggett, Norman A.
Dolga, Amalia M.
Dorandeu, frederic
D'Ovidio, Renato
Dowell, Floyd E.
Dufresne, Marie
Duke, Stephen
Dunière, Lysiane
Dziga, Dariusz
Edwards, Simon
Eller, Kathrin
El-Shehaw, Rehab
Elsinghorst, Paul W.
Emani, Chandrakanth
Eriksson, Johan
Escriche, Baltasar
Escuredo, Olga
Espiña, Begoña
Everson, Sally
Fach, Patrick
Factor-Litvak, Pam
Feig, Andrew L.
Ferenczi, Szilamér
Ferreras, JosÉ Miguel
Fologea, Daniel
Fox, Jay
Franco, Robert S.
Frantisek, Malir
Fratamico, Pina M.
Fujii, Manabu
Fusco, Vincenzina
Galaverna, Gianni
Garcia-Robles, Inmaculada
Gerwin, Robert
Ghareeb, Khaled
Gibbons, John
Gibson, John S.
Gilbert, Robert J.C.
Gilliam, Lyndi L.
Glorieux, Griet
Goldstein, Steve
Gong, Yun Yun
Gorman, Adrienne
Grauso, Laura
Griffiths, Gareth D.
Gromadzka, Karolina
Gruber, Christian
Gryspeirt, Aiko
Guedes, Alonso G.P.
Guerre, Philippe
Gutiérrez, S.
Hadjipanayis, Costas G.
Hall, Roy
Hamada, Masahiko
Hamilos, Daniel L.
Harrison, Robert
Hassan, Fathi
Hassan, Kamal S.
Hatano, Tsutomu
Hayakawa, Tohru
Hayat, Akhtar
Hayes, Fimbarr
Hayes, William
Heasley, Brian H.
Hefter, Harald
Heidt, S.
Hellmann, Nadja
Hellmich, Rick
Helmann, John D.
Hendrich, Suzanne
Hendriksen, Peter J.M.
Hernandez-Alcoceba, Ruben
Herring, Susan
Herzig, Volker
Hippler, Rainer
Hirai, Hirofumi
Hirano, Tsutomu
Hodge, David R
Hodgson, Wayne C.
Hodoki, Yoshikuni
Hofmann, Alan F.
Honkannen, Richard
Hooft, Jamie M.
Hoogland, Govert
Horrigan, Louise A.
Hruska, Zuzana
Hu, Guochang
Hu, Wei-Gang
Huang, Beibei
Huang, Tur-Fu
Huang, Wei-ning
Huang, Yadong
Hudak, Katalin
Hudnell, Kenneth H.
Huertas-Pérez, José F.
Hukriede, Neil A.
Hung, Dong-Zong
Iatsenko, Igor
Ibrahim, Salam A.
Iijima, Noriaki
Iiyama, K.
Ilchmann, Kai
Iliev, Asparouh
Imhof, Diana
Inoue, Seiji
Ippoliti, Rodolfo
Isbister, Geoffrey K.
Ishii, Akira
Isman, Murray B.
Jakka, Siva Rama Krishna
Jančula, Daniel
Jaynes, William
Jean, Ludovic
Jenner, Ronald A.
Jennings, Paul
Jex, Aaron R.
Jjemba, Patrick K.
Joannis-Cassan, Claire
Johansson, Anders
Johler, Sophia
Johnson, Rudolph C.
Juan Domingo, Lieutenant Colonel
Juan, Cristina
Juillerat-Jeanneret, Lucienne
Jungo, Florence
Juttner, Friedrich
Kahlert, Stefan
Kalb, Suzanne R.
Kale, Shubha
Kalinke, Ulrich
Kalkum, Markus
Kang, Hwan-Goo
Kaplan, Bernard S.
Karlovsky, Petr
Karlsson, Oskar
Katayama, Hideki
Katoch, Rajan
Katsoris, Panagiotis
Kawakami, Koji
Kazan, Kemal
Keane, Orla M.
Kędzierska, Barbara
Keller, Nancy
Keller, Ullrich
Kelly, Megan A.
Kersten, Susanne
Keyler, Daniel E.
Kim, Baek-Ho
Kim, Bumseok
Kim, Sung Woo
Kitadokoro, Kengo
Klimaschewski, Lars
Klint, Julie
Koch, Matthias
Kokotos, George
Komori, Yumiko
Kong, Siu-Kai
König, Mario
Koppen, Robert
Kostrzynska, Magdalena
Kouadio, James Halbin
Kowalewski, M.P.
Krasowski, Matthew D.
Krenzelok, Edward P.
Krepkiy, Dmitriy V.
Krienitz, Lothar
Krüger, Thomas
Kudryashov, Dmitri S.
Kulka, Marianna
Kurosawa, Shinichiro
Kuyucak, Serdar
Kuzdraliński, Adam
Kwakkenbos, Mark
Laganà, Aldo
Langley, Ricky L.
LaPlace-Treyture, Christophe
Lecollinet, Sylvie
Ledizet, Michel
Lee, Jeongmi
Lee-Huang, Sylvia
Legname, Giuseppe
Leipold, Enrico
Leistner, Eckhard
Leppla, Stephen H.
Leunert, Franziska
Lévesque, Céline M.
Li, Caixia
Li, Mian
Li, Xiaqing
Liabeuf, Sophie
Liaw, Chih-Chuang
Lin, Hsu Y.
Lind, Owen
Lindkvist-Petersson, Karin
Linstedt, Adam D.
Linz, J.E.
Liu, Julia J.
Liu, Shing-Hwa
Liu, Ying
Lizard, Gérard
Loboda, Agnieszka
Løes, Anne-Kristin
Logan, Susan M.
Loiseau, Nicolas
Lomonte, Bruno
López de Cerain, A.
Lopot, František
Louis, Nancy A.
Lourenço, Wilson R
Luconi, Michaela
Luo, Meng
Lurling, Miquel
Maddau, L.
Malik, Muhammad Arif
Malouin, François
Mancheño, José
Manderville, Richard A.
Mañes, Jordi
Manganelli, Maura
Manning, Bruce
Marceau, François
Maria Gutiérrez, Jose
Marie, Benjamin
Marin, Daniela E.
Marín, Sonia
Markaki, Panagiota
Markland, Frank
Marsche, Gunther
Martin-Eauclaire, Marie-france
Marty, Jean-Louis
Maruyama, Masugi
Maruyama, Toru
Marzocco, Stefania
Matsui, Taei
Mattei, Cesar
Matten, Sharlene R.
Maynard, Jennifer A.
McClain, Mark S.
McClane, Bruce A.
McCormick, John
McCormick, Susan
Mcgrail, Maura
McNeil, Rebecca B.
Meca, Giuseppe
Meijers, Björn
Meinhart, Anton
Melloni, Edon
Melton-Celsa, Angela
Merrill, Rod A.
Meulenberg, Eline P.
Miller, Aaron W.
Miller, Ingrid
Minervini, Fiorenza
Mirey, Gladys
Mitrovic, Simon
Miyake, Shiro
Miyazaki, H.
Moar, William J.
Mochizuki, Naoki
Moeller, Peter D.R.
Moffit, Michelle C.
Mok, Young Sun
Moon, Yuseok
Morales, Michael J.
Moran, C.A.
Moran, Yehu
Morita, Kyoji
Morton, Steve L.
Munkvold, Gary
Murch, Simon H.
Muyldermans, Serge
Myc, Andrzej
Nagashima, Hitoshi
Nagata, Koji
Naidoo, Nirinjini
Narva, Kenneth
Nashan, Björn
Nathanail, Alexis V.
Nees, Matthias
Nelson, Rebecca J.
Nemunaitis, John
Neri, Cristina
Nestorovich, Ekaterina
Nevalainen, Timo
Ng, Kenneth K.S.
NG, Tzi Bun
Nicolas, Aubrey
Nishiwaki, Hisashi
Nishizawa, Kazuhisa
Noguchi, Tamao
O’donnell, Kerry
O’Connor, Sarah
Okumura, Shiro
Onduka, Toshimitsu
Ono, Mario Augusto
Orth, Joachim H.C.
Ossiprandi, Maria Cristina
Otten, Timothy
Ouahid, Youness
Pacheco, Sabino
Paerl, Hans W
Palma, Leopoldo
Pan, Fan
Panaccione, Daniel G.
Pappas, A.C.
Pardee, Angela
Parente, Augusto
Park, Hyun-Woo
Park, Jae H.
Pascale, Michelangelo
Pasquali, Matias
Pauchet, Yannick
Pauron, David
Pawlak, Dariusz
Payan-Carreira, Rita
Payne, Gary
Pérez-Guerrero, Sergio
Persico, Antonio M.
Petricevich, V.I.
Pflugmacher, Stephan
Philbey, A.W.
Pierce-Shimomura, Jon
Pietsch, Constanze
Pinchuk, Iryna
Pincus, Seth
Pinkse, Martijn W.H.
Pinton, Philippe
Piotrowska, Małgorzata
Plane, Frances
Pletinck, Anneleen
Poesen, Ruben
Pohl, Mary Ann
Polak-Jonkisz, Dorota
Poniedziałek, Barbara
Ponikau, Jens U.
Popescu, Ruxandra
Popoff, Michel
Pozzebon, Alberto
Praetorius, Helle A.
Prosperini, Alessandra
Puertollano, Rosa
Py, Sandrine
Quesada, Antonio
Quinn, J.C.
Quinn, Alexander T.
Ramirez, Mário
Rampersad, Joanne
Rash, Lachlan
Ratner, Adam
Ray, Rumiana V.
Recio, Maria C.
Rediske, Richard R.
Reverberi, Massimo
Rhodes, Jonathan
Ribeiro, Bergmann
Richardson, Michael K.
Rigoni, Michela
Rigosi, Anna
Ringe, Dagmar
Robin, Joël
Rodrigues, Paula
Rodriguez de la Vega, Ricardo
Roelants, Kim
Rosenberg, Yvonne
Rosentrater, Kurt
Rossi, Filippo
Rossi, Giuseppe
Rozhon, Wilfried
Ruberson, John R.
Rubert, Josep
Ruiu, Luca
Ruiz, Inigo
Ryu, Dojin
Rzymski, Piotr
Sabatier, Jean-Marc
Sainis, Ioannis
Saint-Cyr, Manuel J.
Sakuda, Shohei
Salmaso, Nico
Sanchez, Joaquin
Sanny, Charles G.
Sardesai, Niranjan Y.
Sarkar, Nurul H.
Schäfer, Wilhelm
Schmidt, Boris
Schmidt, Edward E.
Schmidt, Gudula
Schmitz, Gerd
Schnug, Ewald
Scholz, Bettina
Schrenk, Dieter
Schweiger, Wolfgang
egvić Klarić, Maja
Seifert, Steven A.
Sendall, Barbara
Servin, Alain L.
Sharma, Virender K.
Shaw, Pang-Chui
Shen, Yuh-Chiang
Shinada, Tetsuro
Shinohara, Mari L.
Silva, Christopher J.
Simões, Nelson
Simpson, David M.
Simpson, Tom
Sjöholm, Åke
Smith, Laura E.
Smith, Leo
Solfrizzo, M.
Song, Suquan
Sorlini, Sabrina
Spisni, Alberto
Srinivasan, Jagan
Stanker, Larry H.
Stathopoulos, Christos
Stefanutti, Claudia
Steiner, Ulrike
Stiborova, Marie
Stiles, Brad
Stirpe, Fiorenzo
Stokes, Amber N.
Sturm, Gunter J.
Sugai, Motoyuki
Sukenik, Assaf
Summers, David K.
Suzuki, Hodaka
Suzuki, Toshiyuki
Swearengen, James
Swevers, Luc
Tadahiro, Suzuki
Takacs, Zoltan
Takaya, Yoshiaki
Tanaka, Yoshikazu
Tang, Chih-Hsin
Tateo, F.
Tay, Samuel Sam Wah
Taylor, Bruce
Taylor, Matthew C.
Tester, Patricia
Teter, Ken
Tetreau, Guillaume
Thomas, Christopher
Thorsen, Line
Tiwari, Vaibhav
Tomas, Wood
Tomljenovic, Lucija
Tooker, John
Torres, Jaume
Torres, Victor
Torto, Baldwyn
Townley, Helen E.
Trachtman, Howard
Tran-Dinh, Nai
Trapp, Ralf
Truong, Daniel
Tsuge, Hideaki
Tsushima, Seiya
Tudzynski, Paul
Uchiumi, Toshio
Urriola, Pedro E.
Vale, Paulo
Valenti, Giovanni
Van Frankenhuyzen, Kees
Van Groen, Thomas
Van Leyen, Klaus
Varnum, Susan
Vasconcelos, Vitor
Vassaux, Georges
Verbrugghe, Elin
Vetter, Irina
Vidal, Juan
Vilholm, Ole Jakob
Visser, Petra
Vonk, Freek
Vouros, Paul
Wada, Yoh
Walker, Mary
Wang, Jianqiao
Ward, Marie-Louise
Watanabe, Hiroshi
Weiss, John H.
Wickenden, Alan
Wickramasinghe, Wasantha
Wieczorek, Piotr
Wiegand, Claudia
Wilson, Brenda A.
Wingert, Rebecca A.
Wirth, Margaret
Woloshuk, Charles P.
Wood, Susie A.
Worek, Franz
Woychik, Nancy A.
Woźny, Maciej
Wu, Chih-Jen
Wu, Jin Chuan
Wu, Sheng-Nan
Wu, Vin-Cent
Wu, Yingliang
Yamaguchi, Yoshihiro
Yamamoto, Yoshimasa
Yamashita, Mari Yotsu
Yamazaki, Masahito
Yang, Xuanming
Yao, Jianxiu
Yeste, Marc
Yli-Mattila, Tapani
Yokoyama, Takashi
Yoshihiro, Yamaguchi
Yoshimitsu, Takehiko
Yoshinari, Tomoya
Young, Carolyn A.
Zamarin, Dmitriy
Zamyadi, Arash
Zecconi, Alfonso
Zeng, Weiguang
Zentek, Jürgen
Zhang, Kai
Zhdanov, Alexander V.
Zhorov, Boris S.
Zhou, Ting
Zhou, Xuguo
Ziková, Andrea
Zinkernagel, Annelies S.

